# Oral immunization with a recombinant Lactococcus lactis expressing HIV-1 Gag on the tip of the pilus induces strong mucosal immune responses

**DOI:** 10.1186/1742-4690-9-S2-O12

**Published:** 2012-09-13

**Authors:** V Chamcha, JR Scott, R Amara

**Affiliations:** 1 Emory University, Atlanta, GA, USA

## Background

The induction of a potent humoral and cellular immune response both in the peripheral and mucosal tissues is important for the development of an effective HIV vaccine. The present study explores the ability of a Lactococcus lactis (L. lactis), a probiotic organism, based oral vaccine to elicit HIV-specific immune responses in the mucosal and systemic compartments of mice and rhesus monkeys.

## Methods

To express the HIV-1, Gag (p24) on the tip of the pilus, we made a fusion construct of Gag and Cpa protein of the type-3 pilus found in Streptococcus pyogenes and expressed in L. lactis (rLL-Gag). Four monthly intragastric immunizations were given to the mice and macaques. In mice, we studied the humoral, cellular, and innate immune responses in mucosal and systemic compartments. In macaques, we completed analyses only on the cellular immunity.

## Results

In mice, we observed a strong Gag specific IgG and IgA in serum, feces, and vaginal secretions following rLL-Gag oral immunizations. However, the Gag-specific CD8 T cell responses in the blood were at or below our detection limit. Following an intramuscular MVA/Gag boost, we observed a strong Gag-specific CD8 T cell responses both in systemic and mucosal tissue including IEL/LP of small intestine, peyer’s patches, and mesenteric lymph node. Consistent with immunogenicity, rLL-Gag induced a strong activation of CD8α+, CD11b+ DC in the peyer’s patches at 8 hours after oral immunization. Interestingly, the DC activation was not observed with L. lactis without the pilus. In rhesus macaques, vaccination with rLL-Gag elicited a strong Gag-specific CD4 T cell response both in the blood and rectum producing IL-2 and TNFα and moderate levels of IFNγ and IL-17.

## Conclusion

These results demonstrate that L. lactis expressing antigen on the tip of the pilus can serve as an excellent priming vector to induce a strong mucosal antibody and cellular immunity.

